# Silk–Inorganic Nanoparticle Hybrid Hydrogel as an Injectable Bone Repairing Biomaterial

**DOI:** 10.3390/jfb14020086

**Published:** 2023-02-02

**Authors:** Liangyan Sun, Minqi Lu, Ling Chen, Bingjiao Zhao, Jinrong Yao, Zhengzhong Shao, Xin Chen, Yuehua Liu

**Affiliations:** 1 Department of Orthodontics, Shanghai Stomatological Hospital & School of Stomatology, Department of Macromolecular Science, Fudan University, Shanghai 200433, China; 2 Shanghai Key Laboratory of Craniomaxillofacial Development and Diseases, Fudan University, Shanghai 200001, China; 3 State Key Laboratory of Molecular Engineering of Polymers, Laboratory of Advanced Materials, Fudan University, Shanghai 200433, China

**Keywords:** silk fibroin, nanoparticles, injectable hydrogel, bone regeneration, osteogenesis

## Abstract

Silk fibroin is regarded as a promising biomaterial in various areas, including bone tissue regeneration. Herein, Laponite^®^ (LAP), which can promote osteogenic differentiation, was introduced into regenerated silk fibroin (RSF) to prepare an RSF/LAP hybrid hydrogel. This thixotropic hydrogel is injectable during the operation process, which is favorable for repairing bone defects. Our previous work demonstrated that the RSF/LAP hydrogel greatly promoted the osteogenic differentiation of osteoblasts in vitro. In the present study, the RSF/LAP hydrogel was found to have excellent biocompatibility and significantly improved new bone formation in a standard rat calvarial defect model in vivo. Additionally, the underlying biological mechanism of the RSF/LAP hydrogel in promoting osteogenic differentiation of bone marrow mesenchymal stem cells (BMSCs) was extensively explored. The results indicate that the RSF/LAP hydrogels provide suitable conditions for the adhesion and proliferation of BMSCs, showing good biocompatibility in vitro. With the increase in LAP content, the alkaline phosphatase (ALP) activity and mRNA and protein expression of the osteogenic markers of BMSCs improved significantly. Protein kinase B (AKT) pathway activation was found to be responsible for the inherent osteogenic properties of the RSF/LAP hybrid hydrogel. Therefore, the results shown in this study firmly suggest such an injectable RSF/LAP hydrogel with good biocompatibility (both in vitro and in vivo) would have good application prospects in the field of bone regeneration.

## 1. Introduction

The prevalence of naturally occurring alveolar bone dehiscence and fenestration is high in different kinds of malocclusions [1,2,3,4,5]. These alveolar bone defects may pose potential risks for orthodontic treatment, such as gingival recession and additional bone loss [6,7,8]. These detects can be detected by cone-beam computed tomography (CBCT) before treatment with relatively high accuracy [9,10]. However, effective treatment options, such as periodontally accelerated osteogenic orthodontics (PAOO) and piezocision, require flap turnover or gingival incision [11,12], which are still traumatic and invasive for relatively small and limited defects. In addition, to achieve effective bone tissue regeneration, autologous or allogenic bone graft therapy remains the front line treatment, but it needs an additional donor site and cost. Moreover, its success rate is limited by inherent disadvantages such as infection and donor site morbidity [13,14,15,16]. Nowadays, injectable hydrogel with good biocompatibility has received great attention in the field of bone regeneration as it can be injected and re-expanded to the desired shape in alignment with the defect and serve as an extracellular matrix (ECM) mimic providing a suitable environment for cell growth.

Silk fibroin, a natural protein harvested from the silk of the *Bombxy mori* silkworm, has become a potential candidate for bone tissue regeneration due to its numerous advantages, such as abundant supply, controllable biodegradability, tunable mechanical properties, and excellent biocompatibility [17,18,19,20,21,22]. Therefore, silk fibroin-based materials have been widely applied in tissue engineering, such as scaffolds [21], fibers [23], and hydrogels [24]. However, silk fibroin itself shows insufficient osteoconductive properties, hindering its application in bone tissue engineering. Therefore, researchers always incorporate some other bioactive materials into regenerated silk fibroin (RSF) to solve this problem. For example, Ding et al. used water-dispersible hydroxyapatite (HAp) nanoparticles to prepare an RSF/HAp scaffold for optimized bone repair [21]. Like HAp, Laponite^®^ (LAP, Na^0.7+^[(Mg_5.5_Li_0.3_)Si_8_O_20_(OH)_4_]^0.7−^) is an inorganic nanomaterial, which can also be regarded as a potential candidate for bone defect repairing. LAP is a disc-shaped synthetic smectic clay material with a diameter of 25 nm and a thickness of 1 nm [25]. Recently, many studies have shown that materials containing LAP can promote the osteogenic differentiation of mesenchymal stem cells as well as bone regeneration [26,27,28]. The degradation products of LAP under physiological conditions are non-toxic magnesium ions, silicic acid, and lithium ions, which can further promote cell adhesion and stimulate osteogenesis [26,29,30]. Therefore, the incorporation of LAP nanoplatelets into injectable RSF hydrogel holds promise for repairing bone defects.

In our previous research, an RSF/LAP hydrogel was found to promote the osteogenic differentiation of primary osteoblasts in vitro [31]. However, the promotion effect of RSF/LAP hydrogel on osteogenic differentiation was not fully understood. Herein, the in vivo biocompatibility and osteogenic capability of the RSF/LAP hydrogel were extensively studied. BMSCs are essential for bone regeneration due to their osteogenic differentiation potential [32,33], and studies have shown that BMSC-based approaches had a favorable effect on bone formation [34]. Hence, the effect of BMSCs on the osteoblastic differentiation was evaluated to explore the underlying biological mechanism of the RSF/LAP hydrogel.

## 2. Materials and Methods

### 2.1. Preparation of RSF/LAP Hydrogel

*B. mori* silkworm cocoons were purchased from Jiangsu Province, China. LAP was kindly provided by Donghua University, China. RSF aqueous solutions were obtained, following previously established methods of this laboratory. Briefly, *B. mori* silkworm cocoons were degummed in an aqueous solution of 0.02 mol L^−1^ Na_2_CO_3_ at 100 °C for 45 min, rinsed thoroughly with deionized water three times, and then dried at 40 °C. Afterwards, the degummed silk was dissolved in 9.3 mol L^−1^ LiBr solution at 45 °C for 1 h and dialyzed against deionized water for 3 days using a 12–14 kDa cutoff dialysis membrane to remove salts. After centrifugation at 8000 r min^−1^ (~5000× *g*) for 10 min, the acquired RSF aqueous solution with a concentration of approximately 4.5% (*w*/*w*) was stored at 4 °C for further use.

LAP powder was first dispersed in water under stirring for 24 h and then mixed with RSF solution. The final RSF concentration was adjusted to 4% (*w*/*w*), and the LAP content was 0, 1, 3, or 5% (*w*/*w*) relative to the silk fibroin. The mixture was then sonicated for 2 min at 60% amplitude using a FS-1200N sonifier (1200 W, SX-Sonic. Co., Shanghai, China) and subsequently incubated at 37 °C. Inverted test tube method was adopted to monitor the sol–gel transition. The RSF/LAP hydrogel samples were labeled as RSF/1%LAP, RSF/3%LAP, and RSF/5%LAP according to the LAP content of 1, 3, or 5% (*w*/*w*) relative to the silk fibroin.

### 2.2. Characterization of RSF/LAP Hydrogel

Morphology of the RSF/LAP hydrogel was examined under a field emission scanning electron microscope (FE-SEM, Ultra 55, Zeiss, Oberkochen, Germany). Prior to observation, the hydrogel was lyophilized and then sputter-coated with gold. Rheological tests were performed at 37 °C in strain-controlled mode using a Physica MCR 301 rheometer (Anton Paar GmbH, Graz, Austria) with 25 mm parallel-plate configuration and 1 mm gap distance. The RSF/LAP hydrogel (1 mL) was dispensed on the bottom plate, and low density mineral oil was poured around the hydrogel to minimize evaporation. Dynamic frequency sweep experiments of the RSF/LAP solution and hydrogel were performed in the range of 100–0.1 rad s^−1^ at 1% strain. For the shear recovery test, the RSF/LAP hydrogel was subjected to 0.1% strain, followed by 1000% strain for 30 s, and finally returned to 1% strain with 1 rad s^−1^ frequency. Such cyclic tests were repeated seven times. All the hydrogels were sterilized with γ-irradiation at a dose of 25 kGy and tiled on the wells or filled syringes for subsequent experiments.

### 2.3. In Vivo Biocompatibility

All animal procedures in this study were performed in accordance with the guidelines for care and use of laboratory animals of Fudan University and approved by the animal ethics committee of Fudan University (202206022Z).

In vivo biodegradation and biocompatibility tests were conducted using subcutaneous implantation in Sprague–Dawley (SD) rats. To relieve pain, the rats were anesthetized using intraperitoneal injections of pentobarbital (35 mg kg^−1^). The dorsal hair of the rats was shaved, and the skin was disinfected with iodophor. After being sterilized, 200 μL of freshly prepared RSF/LAP hydrogel was implanted by injection into individual dorsal subcutaneous pockets. After 14 days, the rats were euthanized, and the constructs and surrounding tissue were explanted and analyzed for biocompatibility using histological and immunohistological evaluations.

After being fixed in 4% (*w*/*v*) paraformaldehyde for 24 h, the samples were dehydrated using gradient ethanol solutions (75% (*v*/*v*) ethanol 4 h, 85% (*v*/*v*) ethanol 2 h, 90% (*v*/*v*) ethanol 2 h, 95% (*v*/*v*) ethanol 1 h, anhydrous ethanol I 30 min, anhydrous ethanol II 30 min, and anhydrous ethanol III 30 min), cleared in xylene (xylene I 20 min, xylene II 20 min, and xylene III 20 min), embedded in paraffin (48–50 °C melting paraffin I overnight, 56–58 °C melting paraffin II 2 h, and 60–62 °C melting paraffin III 2 h), and sectioned at a thickness of 4 μm by the paraffin slicer (RM2016, Leica, Wetzlar, German). The tissue was flattened when the slice floated on the 40 °C warm water of the spreading machine, and the tissue was picked up using the glass slides and baked in the oven at 60 °C. After the water-baked dried wax was melted, it was taken out and stored at room temperature. After deparaffinization (xylene I 20 min, xylene II 20 min, xylene III 20 min, anhydrous ethanol I 10 min, anhydrous ethanol II 10 min, anhydrous ethanol III 10 min, 95% (*v*/*v*) ethanol 10 min, 90% (*v*/*v*) ethanol 10 min, 85% (*v*/*v*) ethanol 10 min, 75% (*v*/*v*) ethanol 10 min, rinsing with tap water), they were stained with hematoxylin–eosin (H&E, Servicebio, Beijing, China), Masson trichrome staining (Servicebio), and immunohistochemical staining. Vessel formation of these samples was examined using immunohistochemical staining with anti-CD31 (Abcam, Cambridge, UK), while the inflammatory response was determined based on the presence of inflammatory markers, including CD3 (Abcam) and CD68 (Abcam).

### 2.4. In Vivo Bone Regeneration of the Hydrogels

To evaluate the in vivo bone forming ability of the hydrogels, a rat critical-sized calvarial defect model was used. Twenty-four 8-week-old male SD rats (180–200 g) were used and randomly divided into four groups: blank, RSF, RSF/1%LAP, and RSF/5%LAP. The rats were anesthetized using intraperitoneal injections of pentobarbital (35 mg kg^−1^). Afterward, a 1.5 cm sagittal incision was made on the scalp to expose the calvarium by blunt dissection. Then, critical-sized calvarial bone defects (5 mm diameter) were created on two sides of the parietal bone. After each defect was filled with the pristine RSF and RSF/LAP hybrid hydrogels, the incision was closed in layers using resorbable sutures. After surgery, the rats were kept in pathogen-free (SPF) laboratory animal room and fed. At 8 and 12 weeks, the rats were euthanized using intraperitoneal injections of pentobarbital overdose (150 mg kg^−1^) to obtain specimens.

After fixing with 4% (*w*/*v*) paraformaldehyde for 24 h, microcomputed tomography (micro-CT, SkyScan 1272, SkyScan, Aartselaar, Belgium) of the cranial specimens was performed to evaluate the bone formation within the bone defects. The scanning parameters were as follows: voltage 70 kV, current 142 μA, and resolution 18 μm. The collected 3D scans were reconstructed and analyzed using CTan (SkyScan). After micro-CT imaging and analysis, the harvested samples were sequentially decalcified with 10% (*w*/*v*) EDTA, dehydrated through gradient ethanol solutions, and embedded in paraffin. Then, 4 μm thick sections were used for hematoxylin–eosin and Masson trichrome staining. Furthermore, immunohistochemistry targeting with osteocalcin (OCN) (Abcam) and osteopontin (OPN) (Abcam) was used to reveal new bone formation at defect sites.

### 2.5. BMSC Isolation and Identification

BMSCs were extracted from male SD rats (40 g) according to the method reported in the literature [35] and cultured in α-MEM culture medium containing 15% (*v*/*v*) FBS (Gibco, NY, USA) and 1% (*v*/*v*) penicillin/streptomycin (100×, Gibco) at 37 °C and under 5% (*v*/*v*) CO_2_. The media were changed every 3 days, and third generation BMSCs were used in subsequent experiments.

Flow cytometric analysis was performed to identify the presence of MSC-positive (CD29 and CD90) and MSC-negative (CD45) surface markers using the appropriate antibodies (Biolegend, San Diego, CA, USA). Alizarin Red S staining after osteogenic induction and Oil Red O staining after adipogenic induction were performed to identify the stemness of the cells [36].

### 2.6. In Vitro Biocompatibility

BMSC suspension was prepared with a concentration of 5 × 10^4^ mL^−1^ after cell digestion and centrifugation.

Biocompatibility of the RSF/LAP hydrogel was evaluated using the Cell Counting Kit-8 (CCK-8) test. In total, 100 μL corresponding hydrogels were spread in 96-well plates, and then 100 μL cell suspension was added into each well. BMSCs were cultured on the surface of pristine RSF hydrogel and RSF/LAP hydrogels. After 1, 4, and 7 days, the samples were washed with PBS solution and incubated in CCK-8 along with cell culture medium in a ratio of 1:10. After incubation for 2 h, 100 μL supernatant was transferred into new 96-well culture plates, and the absorbance of the solution at 450 nm was measured using a microplate reader (Epoch2, Biotek, Winooski, VT, USA).

After culturing for 1, 4, and 7 days, the cellular attachment and spreading of BMSCs on the surface of the hydrogels was recorded. On days 1 and 7, BMSCs on the RSF/5%LAP hydrogels were washed three times with PBS and fixed with 4% (*w*/*v*) paraformaldehyde for 15 min at room temperature. After permeabilization with 0.1% (*v*/*v*) Triton X-100 (Sigma, St. Louis, MO, USA) for 10 min, the cell actin and nuclei were stained with fluorescein isothiocyanate (FITC)–phalloidin (Thermo Fisher, Waltham, MA, USA) and 4’,6-diamidino-2-phenylindole (DAPI). Fluorescence images of BMSCs were obtained using a confocal microscope (CLSM, Nikon C2+, Tokyo, Japan). To further investigate the morphology of the cells in the hydrogel, after culturing for 7 days, the samples were washed 3 times with PBS and fixed in 2.5% (*v*/*v*) glutaraldehyde (4 °C) overnight. After they were washed 3 times with PBS, the samples were post-fixed with 1% (*w*/*v*) OsO4 (4 °C) for 2 h, washed 3 times with PBS, and dehydrated using graded ethanol solutions (50% (*v*/*v*) ethanol 15 min, 70% (*v*/*v*) ethanol 15 min, 90% (*v*/*v*) ethanol 15 min, anhydrous ethanol I 15 min, anhydrous ethanol II 15 min, and anhydrous ethanol III 15 min). Then, they were dried with CO_2_ at critical point (31 °C and 74 bar) in a critical point dryer (HCP-2, Hitachi, Tokyo, Japan) for 2 h, underwent vacuum coating for 3 min (EMAG600, Leica) with the inoculation side up, and observed under FE-SEM (SU8010, Hitachi).

### 2.7. In Vitro Osteogenic Property Analysis

BMSC suspension was prepared using a concentration of 10^5^ mL^−1^ after cell digestion and centrifugation. ALP activity, ALP staining, Alizarin Red staining, and bone-related gene and protein expressions were assessed to evaluate the osteogenic differentiation ability of BMSCs.

For ALP activity assay and bone-related gene and protein expression analysis, the hydrogels with a thickness of 2 mm were first spread at the bottom of the culture plates, and then BMSCs were cultured directly on the pristine RSF hydrogel and RSF/LAP hydrogels in culture plates. For ALP staining and Alizarin Red staining, 0.5 mL hydrogel was carefully injected into 6-well transwell plates and co-cultured with BMSCs. After 1 day of culture, the medium was changed to osteogenic differentiation medium with osteoinductive factors and then changed every 3 days.

For ALP activity quantification, cells were harvested on days 7 and 14. An ALP assay kit (Beyotime, Shanghai, China) was applied to determine the ALP activity following the manufacturer’s instructions. The absorbance at 405 nm was recorded, and the ALP value was obtained according to the standard curve plotted by the standard samples. Bicinchoninic acid (BCA) protein assay kit (Pierce, IL, USA) was applied to evaluate the total protein levels. ALP activity was normalized to the total protein concentration of the samples. ALP staining was performed on day 7 using a BCIP/NBT alkaline phosphatase color development kit (Beyotime, China) according to the manufacturer’s protocol.

Alizarin Red staining was performed on day 21. Samples were gently rinsed with distilled water and fixed with 4% (*w*/*v*) paraformaldehyde for 10 min at room temperature. Calcium accumulations of the samples were stained with a solution of 2% (*v*/*v*) Alizarin Red S (Sigma-Aldrich, St. Louis, MO, USA) for 10 min, then they were washed thoroughly with PBS and photographed. Quantitative analysis of Alizarin Red staining was performed by eluting the bound stain with 10% (*w*/*v*) cetylpyridinium chloride (Sigma-Aldrich). The absorbance of the resulting solution at 562 nm was measured using a microplate reader (Epoch2, Biotek, Winooski, VT, USA).

On day 7, RNA of BMSCs cultured on the RSF/LAP hydrogels was extracted using Trizol reagent and quantified using UV-Vis spectrophotometer (NanoDrop 2000, Thermo, Waltham, MA, USA), then cDNA was synthesized using 1 μg of total RNA. Real-time qPCR was conducted with SYBR Green Mix using a thermal cycler (LightCycler 96, Roche, Basel, Switzerland). Before reaction, 20 μL of reaction volume was mixed, including 10 μL of 2 × SYBR Green (Super Real PreMix Plus, Tiangen, Beijing, China), 0.3 μL of each primer (100 μM), 1 μL of cDNA, and 8.4 μL of sterile distilled water, which was amplified at 95 °C for 1 min and then 40 cycles of 95 °C for five seconds, followed by 60 °C for 30 s. Gene expression levels of the target genes (Runt-related transcription factor 2 (Runx2), collagen type I (Col1), OPN, and OCN) were evaluated in triplicate. The 2^−ΔΔCt^ method was used, and the results were calculated and normalized to the level of β-actin. The relevant primer sequences are listed in Table S1.

Western blot analysis was performed to determine the expression levels of bone morphogenetic protein 2 (BMP-2), Runx2, and osterix (OSX), with β-actin as the internal control. After being cultured on the hydrogels for 14 days, total proteins were extracted from the cells by lysing in RIPA buffer containing a protease inhibitor cocktail and then treated with protein loading buffer at 95 °C for 10 min. Protein concentration was measured using BCA protein assay kit (Pierce, IL, USA). The extracted proteins (20 μg) were separated by SDS-PAGE (120 V, 70 min) and then transferred onto a poly-vinylidene difluoride (PVDF) membrane (250 mA, 60 min). After blocking with 5% (*w*/*v*) skimmed milk in Tris-buffered saline Tween-20 (TBST) for 1 h, the membranes were incubated with primary antibodies specific for BMP-2 (1:1000, Proteintech, Protein, Rosemont, IL, USA), Runx2 (1:1000, Cell signaling technology, Boston, MA, USA), OSX (1:1000, Boster biological technology, Pleasanton, CA, USA), and β-actin (1:2000, Absin, Shanghai, China) at 4 °C overnight. After washing three times in TBST for 5 min, the membranes were incubated with the appropriate horseradish peroxidase-coupled secondary antibodies (1:10,000, Cell Signaling Technology) against the primary antibodies at room temperature for 1 h. Proteins on the membranes were detected using an enhanced chemiluminescent substrate kit (ECL Advance; Thermo Fisher Scientific) and visualized with an imaging system (Amersham Imager 600, General Electric company, Atlanta, GA, USA). The protein band intensity was quantified using ImageJ software 1.52a (National Institutes of Health, Boston, MA, USA).

### 2.8. Mechanism Investigation

After being cultured on RSF/LAP hydrogels with gradient LAP content for 24 h, protein of the BMSCs was extracted for Western blot analysis to explore the role of LAP in the AKT signaling pathway. To further investigate the role of AKT signaling, 10 μM MK2206 (AKT1/2/3 inhibitor, Selleck, Houston, TX, USA) was used to suppress AKT phosphorylation. Western blot and real-time qPCR analyses were conducted after culturing on the RSF/LAP hydrogels for 24 h, while ALP staining was conducted to verify the osteogenic differentiation of BMSCs cultured for 7 days. The following primary antibodies were used against specific proteins: AKT (1:2000, Proteintech), p-AKT (1:2000, Cell signaling technology), BMP-2 (1:1000, Proteintech), and β-actin (1:2000, Absin). The experimental protocols of Western blot, real-time qPCR, and ALP staining were the same as previously described.

### 2.9. Statistical Analysis

Statistical analysis was performed using SPSS 19.0 (SPSS Inc., Chicago, IL, USA). Shapiro–Wilk test and Q-Q plots were used to evaluate the normality of the analyzed data. Differences between multiple or two groups were analyzed using one-way analysis of variance (ANOVA) or Student’s *t*-test, respectively. At least three independent replicates were used for each measurement, and the data were presented as mean ± standard deviation. *p* values < 0.05 were considered statistically significant. Figures were prepared using the GraphPad software 6.0 (GraphPad Software, Inc., San Diego, CA, USA).

## 3. Results and Discussion

### 3.1. Preparation and Characterization of RSF/LAP Hydrogels

**Figure 1:**
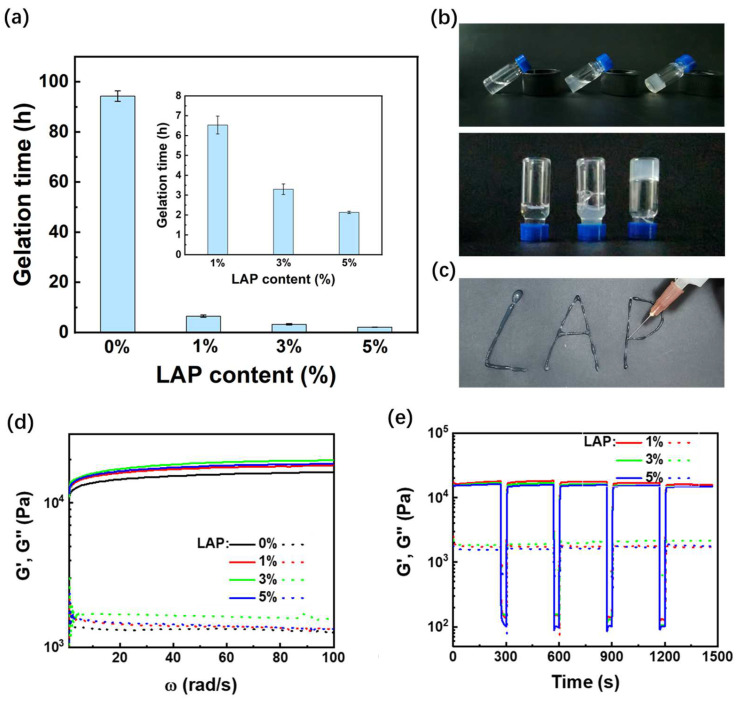
Preparation and characterization of RSF/LAP hydrogels: (**a**) gelation time with different LAP contents; (**b**) images of the hydrogel formation process. From left to right: before sonication, immediately after sonication, and after gelation; (**c**) injectability of the hydrogel; (**d**) frequency sweep of various RSF/LAP hydrogels with 1% strain; (**e**) repetitive recovery test of the RSF/LAP hydrogels.

It is well known that the conformation of silk fibroin can be easily transformed from random coil/helix to β-sheet conformation by varying the temperature and pH conditions, or by shearing or sonication treatments. Herein, RSF/LAP hydrogels were obtained from RSF/LAP aqueous dispersion with a sonication pretreatment for 2 min combined with incubation at 37 °C. Figure 1a shows the gelation time of the RSF/LAP hydrogels with different LAP contents. The introduction of LAP significantly shortened the gelation time, i.e., from ~90 h for pristine RSF hydrogel to ~2 h for the RSF/5%LAP hydrogel (Figure 1b). The fast formation of the RSF/LAP hydrogel is favorable for the homogeneous dispersion of LAP in the matrix of the RSF hydrogel. The EDS elemental mapping shown in Figure S1 confirmed that both Si and Mg elements in LAP were well dispersed in the RSF matrix.

As a thixotropic hydrogel designed for fixing bone defects, the injectable properties of the RSF/LAP hydrogel are of great importance. Figure 1c shows the direct impression of the RSF/LAP hydrogel, which can be easily injected from a needle. Figure 1d presents the general rheological properties of pristine RSF hydrogel and RSF/LAP hydrogels. It can be seen that the storage modulus G′ of the hydrogel increases slightly from about 15.9 for pristine RSF hydrogel to 20.0 kPa for RSF/LAP hydrogels. This suggested that the incorporation of LAP in RSF improved the mechanical properties of the RSF hydrogel. In addition, large amplitude oscillatory shear was employed to evaluate the thixotropic property of the RSF/5%LAP hydrogel. Figure 1e shows that when the RSF/5%LAP hydrogel was subjected to a large strain treatment of 1000%, it rapidly exhibited liquid behavior. However, when the strain was reduced back to 1%, the liquefied RSF/5%LAP hydrogel immediately exhibited gel behavior. When the RSF/5%LAP hydrogel was subjected to 1000% strain for 30 s and returned back to 1% strain, its G′ recovered to at least 90% of its original value within 270 s. Such a cycle could be repeated at least seven times, indicating that the RSF/5%LAP hydrogel can be easily injected into desired forms in practical clinical application.

### 3.2. In Vivo Biocompatibility of RSF/LAP Hydrogels

The excellent potential of such an injectable RSF/LAP hybrid hydrogel in the application of bone defect repair was demonstrated in our previous work mainly via in vitro experiments [31]. To further evaluate the possibility of the RSF/LAP hydrogel in real clinical application, in vivo experiments were performed in this study. First, subcutaneous implantation was applied to evaluate the biocompatibility of the RSF/LAP hydrogel in vivo.

**Figure 2:**
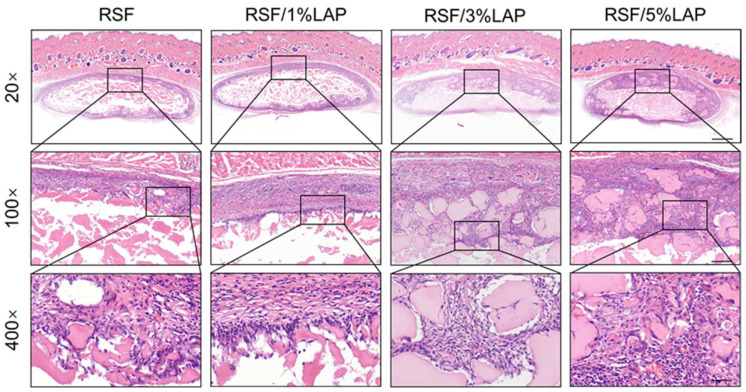
H&E staining images of the RSF/LAP hydrogels after subcutaneous implantation for 2 weeks. Scale bars: 20×, 1 mm; 100×, 200 μm; 400×, 50 μm.

Histological analysis via H&E staining (Figure 2) showed early and progressive ingrowth of tissue from the recipient into the samples, indicating good biocompatibility and integration of the hydrogels in vivo. Although there was no significant difference in the thickness of the remaining hydrogels in different groups at low magnification, the sectional area of the ingrowth tissue of the RSF/5%LAP hydrogel was the largest. It was also found that when the LAP content in the hydrogel was higher, the degradation rate of the RSF/LAP hydrogel was slower. According to the previous study [31], LAP in the RSF hydrogel could both increase the number and limit the growth of the β-sheet domains that served as cross-linkers of the hydrogel. Thus, more β-sheet domains with smaller sizes were formed in the RSF/LAP hydrogel with larger LAP content, which led to a slower degradation rate. Considering the potential application for bone regeneration, the characteristic of relatively slow implant degradation is beneficial for allowing substantial cellular ingrowth and replacement of the hydrogel by new bone [37]. In addition, it is worth mentioning that the degradation products of RSF and LAP are all absorbable and non-toxic [19,26].

**Figure 3:**
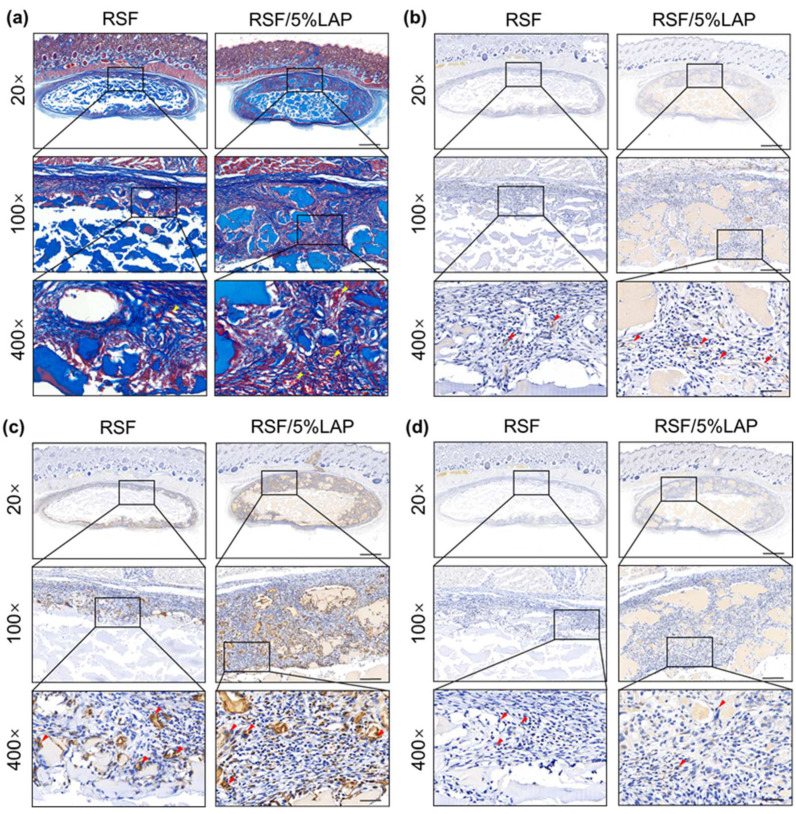
Masson and immunohistochemistry staining images of the RSF/LAP hydrogels after subcutaneous implantation for 2 weeks: (**a**) Masson trichrome staining. The yellow arrows indicate the blood vessels which contain red blood cells; (**b**) immunohistochemistry staining assessed using CD31. The red arrows indicate new vessel formation in the interfacial zone between the hydrogel and the host tissue; (**c**) immunohistochemistry staining assessed using CD68 (macrophages are indicated by red arrows); (**d**) immunohistochemistry staining assessed using CD3 (lymphocytes are indicated by red arrows). Scale bars: 20×, 1 mm; 100×, 200 μm; 400×, 50 μm.

Masson staining and immunohistochemistry staining (CD31) were also conducted to further explore the vessel formation and inflammatory response in the interface zone between the hydrogel and host tissue. The staining results revealed new angiogenesis (pointed at by arrows in Figure 3b) in both groups. In addition, there was mild macrophage invasion (CD68), and a few lymphocytes were detected (CD3), indicating a mild and controllable inflammatory response in the experimental groups either with or without LAP (Figure 3c,d). Therefore, it can be concluded that the RSF/LAP hydrogel possessed good in vivo biocompatibility.

### 3.3. In Vivo Bone Regeneration of the Hydrogels

**Figure 4:**
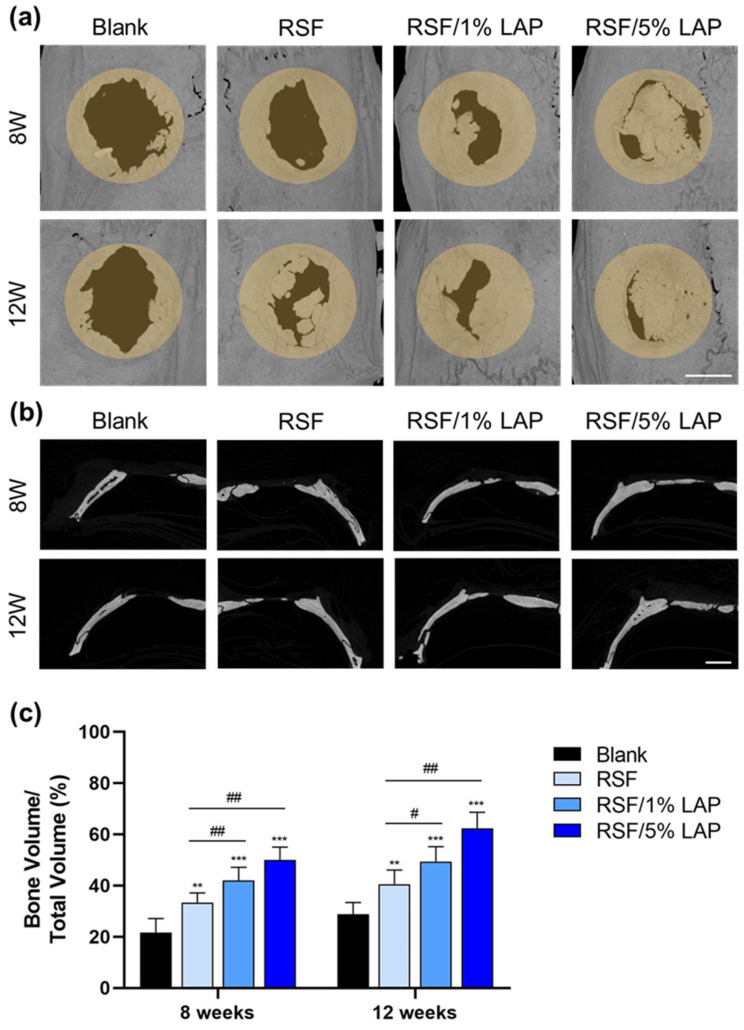
Micro-CT image analysis of new bone formation in a rat calvarium model treated with blank group and different RSF/LAP hydrogels: (**a**) 3D reconstructed images of defect sites at 8 and 12 weeks after surgery. Scale bar, 2 mm; (**b**) 2D images of sagittal sections at 8 and 12 weeks after surgery. Scale bar, 2 mm; (**c**) quantification of BV/TV. ** *p* < 0.01, *** *p* < 0.001. RSF/LAP hydrogels versus pristine RSF hydrogel: # *p* < 0.05, ## *p* < 0.01.

As RSF/LAP hydrogel shows good in vivo biocompatibility, a rat calvarial defect model was used to further assess its osteogenesis capacity. The 3D structure of the defect in the cranium of the rats was investigated using micro-CT, as shown in Figure 4a. For all RSF/LAP hydrogels, the contour of the defect decreased after 8 to 12 weeks of implantation, suggesting a bone healing process. As a critical-sized defect model, the unfilled defects remained void after 12 weeks, while the hydrogel-filled defects showed remarkable tissue regeneration in the peripheral area of the defect. Compared to that of the pristine RSF group, bone regeneration was significantly faster in the RSF/LAP hydrogel group. The best bone reconstruction was achieved in the RSF/5%LAP hydrogel group with the highest LAP content. The 2D images of the sagittal sections also revealed different bone healing processes (Figure 4b). In the RSF/LAP groups, there was obvious new bone formation in the central region of the defect, especially in the RSF/5%LAP hydrogel group at 12 weeks. In contrast, the defects treated with pristine RSF hydrogel remained nonunion in the central region. Quantification analysis of bone volume/total volume (BV/TV) (Figure 4c) exhibited similar trends for different hydrogels, and the best values were achieved for the defects treated with RSF/5%LAP hydrogels. At 8 weeks, the BV/TV percentage values (%) were 21.7 ± 5.0, 33.3 ± 3.5, 42.1 ± 4.7, and 49.9 ± 4.7 with blank, pristine RSF, RSF/1%LAP, and RSF/5%LAP hydrogel samples, respectively. At 12 weeks, the values increased to 28.8 ± 4.2, 40.5 ± 5.1, 49.4 ± 5.3, and 62.3 ± 5.8, respectively. This result was consistent with the report by Miao et al., who used 3D bioprinted LAP hydrogel for bone defect reconstruction [38]. The micro-CT analysis in this study revealed that LAP improved the bone-forming ability of the RSF hydrogel. In the literature, Ding et al. used an RSF-Hap hydrogel for in vivo bone repair [21]. Compared with their BV/TV values, the corresponding values at 8 weeks in this work are higher, while at 12 weeks, the BV/TV values are equivalent. As Hap is the main mineral phase of bone and shows outstanding osteoconductivity in RSF/Hap composites [39], this result firmly suggests that the RSF/5%LAP hydrogel is suitable for in vivo bone regeneration.

**Figure 5:**
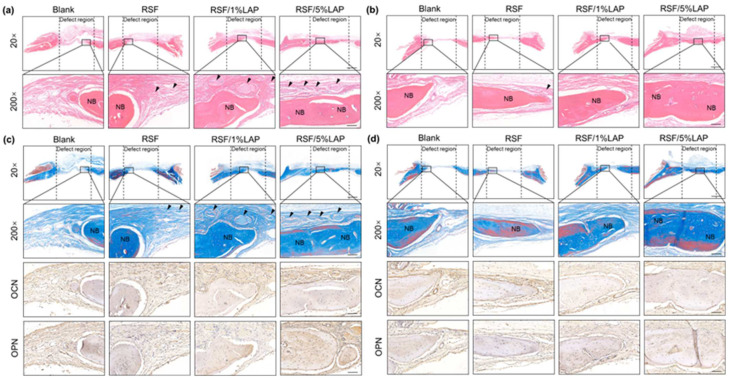
Histological and immunohistochemical staining analysis of bone formation in a rat calvarium model treated with blank group and different RSF/LAP hydrogels: (**a**) H&E staining of the defect area at 8 weeks after surgery; (**b**) H&E staining of the defect area at 12 weeks after surgery; (**c**) Masson trichrome staining and OCN and OPN expressions of the defect at 8 weeks after surgery; (**d**) Masson trichrome staining and OCN and OPN expressions of the defect at 12 weeks after surgery. Black arrows indicate unabsorbed hydrogel; NB shows new formed bone. Scale bars: 20×, 1 mm; 200×, 100 μm.

Histological and immunohistochemical analyses of samples were used to clarify bone reconstruction behavior inside the RSF/LAP hydrogels (Figure 5). H&E staining and Masson trichrome staining images were obtained using a light microscope at various levels of magnification. H&E staining at 8 and 12 weeks showed newly formed osteoid tissue, indicating the formation of new bone inside the hydrogels (Figure 5a,b). Masson trichrome staining results also indicated gradually faster and better bone regeneration at the defects treated with RSF/LAP hydrogels. In the Masson trichrome staining images, margins of the defect were mainly connected by a connective fibrous tissue layer with a small amount of newly formed bone in the RSF group. In the RSF/1%LAP group, the defects were occupied by thick dense fibrous connective tissue with thin bone-like tissue. In contrast, in the RSF/5%LAP group, the defects were almost completely filled with bone-like tissue. In addition, in both the H&E and Masson trichrome staining results, certain amounts of unabsorbed hydrogels were seen after 8 weeks of implantation, while only a few could be detected in the samples after 12 weeks. This result indicates that the hydrogel can remain in the body for over 2 months and gradually be replaced by new bone. The degradation of the RSF/LAP hydrogels after 8 weeks of implantation was slower than that of the pristine RSF hydrogel, especially at higher LAP contents, which was consistent with the in vivo experimental results of subcutaneous implantation. This feature is beneficial for substantial cellular ingrowth and new bone formation by continuously maintaining the space of the bone defect area, recruiting host cells, and releasing ions in LAP that promote bone regeneration. The quality of the regenerated bone in all experimental groups at 8 and 12 weeks was evaluated through immunohistochemical staining of OCN and OPN, two typical markers of osteogenic differentiation and mineralization. As shown in Figure 5c,d, more mature bone tissue was found in the defects when they were treated with the RSF/5%LAP hydrogel. Regenerated bone tissue with richer collagen deposition and better osteoid matrix was achieved in the RSF/5%LAP hydrogel group, which exhibited similar composition and structure to the surrounding natural bones. In summary, these results strongly support that the RSF/5%LAP hydrogel is suitable for in vivo bone repair.

Although the above results demonstrated that the RSF/LAP hybrid hydrogel was effective for in vivo bone repair, the mechanism by which it promotes osteogenic differentiation is still not clear. BMSCs are multipotent progenitor cells localized in the stromal compartment of the bone marrow, which are essential for bone regeneration due to their osteogenic differentiation potential [32,33], and osteocytes are derived from the BMSC lineage through osteoblast differentiation [40]. Therefore, it was assumed that the RSF/LAP hydrogels stimulate bone regeneration by promoting the osteogenic differentiation of resident BMSCs. In the field of bone repairing materials, the promotion of material on the osteogenic differentiation of resident BMSCs is an important factor to evaluate the bone regeneration ability. In vitro experiments were further conducted to verify the biocompatibility of RSF/LAP hydrogels and their effect on the osteoblastic differentiation of BMSCs.

### 3.4. Effect of LAP Addition on In Vitro Biocompatibility of RSF/LAP Hydrogels

Flow cytometric analysis demonstrated that BMSCs were positive for CD29 and CD90 but negative for the hematopoietic cell marker CD45 (Figure S2a). The multipotency of BMSCs was assessed after osteogenic and adipogenic induction and determined using Alizarin Red S (Figure S2b) and Oil Red O (Figure S2c) staining methods, respectively. These cells formed red extracellular mineralized nodules or red lipid droplet clusters under certain conditions, which indicated that the isolated BMSCs possessed multi-differentiation potential identical to stem cells.

**Figure 6:**
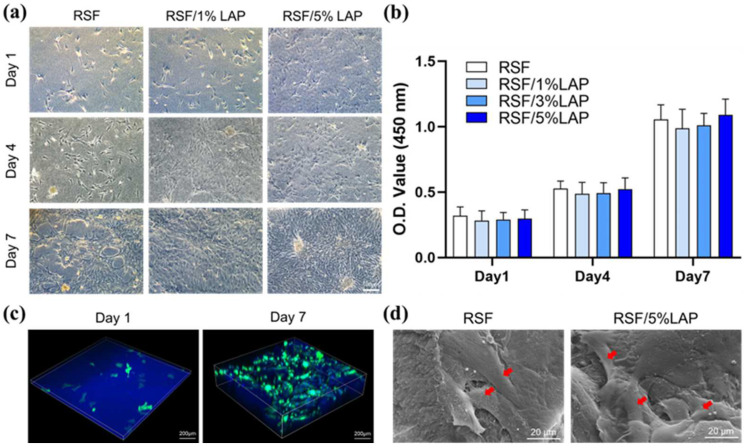
In vitro biocompatibility of the RSF/LAP hydrogels: (**a**) spreading of BMSCs on the surface of the hydrogels; (**b**) proliferation of BMSCs in the hydrogels for 1, 4, and 7 days (detected using CCK-8 analysis); (**c**) three-dimensional confocal images of BMSCs cultured in RSF/5% LAP hydrogel for 1 day and 7 days; (**d**) SEM images of BMSCs in RSF and RSF/5% LAP hydrogels for 7 days (red arrows: spread morphology of BMSCs).

The surface topographies of BMSCs spreading on different RSF/LAP hydrogels were preliminarily analyzed using microscopic observation at different incubation times (Figure 6a). After culturing for 1 day, the cells were attached and distributed evenly on the hydrogels with elongated morphology in all three groups, while after culturing for 7 days, the number of cells in each group increased significantly. The cells showed a good growth state since day 4. Consistently, the CCK-8 assay results demonstrated almost equal viability of BMSCs cultured on the hydrogels with different LAP contents at the same incubation time and revealed a significant increase in cell proliferation after 4 days of culturing (Figure 6b). The confocal microscopy images indicated that BMSCs were not only adhered onto the surface of the RSF/LAP hydrogel but also gradually grew into them (Figure 6c). Many spindle-shaped cells existed inside the hydrogel after culturing for 7 days, suggesting the significant proliferation and good spreading of the cells in different directions (Figure 6c). To further observe the shape and morphology of BMSCs inside the RSF/LAP hydrogels and confirm the effective adhesion of the cells and the material, SEM images were obtained and analyzed. Figure 6d shows typical cell growth in both pristine RSF and RSF/5%LAP hydrogels after incubation for 7 days. It can be seen that BMSCs presented an elongated and flattened morphology fused together on the material with pseudopodia (pointed at by red arrows).

The above experimental results indicated that the RSF/LAP hydrogels provided suitable conditions for the adhesion and proliferation of BMSCs. This is undoubtedly the basis for the osteogenic differentiation of BMSCs, which plays an important role in promoting bone regeneration.

### 3.5. Effect of LAP Addition on In Vitro Osteodifferentiation of BMSCs in RSF/LAP Hydrogels

**Figure 7:**
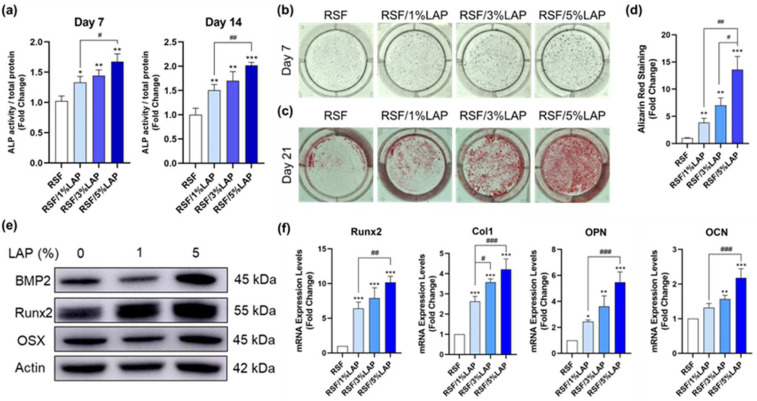
Evaluation of the osteoinductivity of different RSF/LAP hydrogels: (**a**) ALP activity of BMSCs cultured on the hydrogels for 7 and 14 days; (**b**) ALP staining on day 7; (**c**) Alizarin Red staining on day 21; (**d**) Alizarin stain intensity evaluated using densitometry; (**e**) osteogenic-related protein expression of BMSCs (BMP2, Runx2, OSX, and β-actin) on day 14; (**f**) osteogenic-related gene expression of BMSCs (Runx2, Col1, OPN, and OCN) on day 7. * *p* < 0.05, ** *p* < 0.01, *** *p* < 0.001. RSF/LAP hydrogels versus pristine RSF hydrogel: # *p* < 0.05, ## *p* < 0.01, ### *p* < 0.001.

The previous study showed that the involvement of LAP greatly promoted the osteogenic differentiation of primary osteoblasts [31]. In this study, the role of the RSF/LAP hydrogel in promoting the osteogenic differentiation of BMSCs was further investigated, as it is crucially important for bone defect repair. ALP is recognized as an early marker for osteogenic differentiation as it can hydrolyze inhibitors of mineral deposition [41,42]. Figure 7a shows that with the increase in LAP content, the ALP level gradually increased either after 7 or 14 days of incubation. The ALP staining results displayed in Figure 7b show the same trend. The intensity of mineralized extracellular matrix was assessed using Alizarin Red staining (Figure 7c) and quantitation (Figure 7d) after 21 days of incubation. The highest level of calcium deposition was observed in the RSF/5%LAP hydrogel group, suggesting that the RSF/5%LAP hydrogel was ideal for bone regeneration approaches. In addition, the gene expression levels of osteogenic markers were analyzed, and the results are shown in Figure 7e. In comparison with the pristine RSF hydrogel, gene expressions of Runx2, Col1, OPN, and OCN were significantly increased in the RSF/LAP hydrogel groups, especially for RSF/5%LAP with the highest LAP content. Moreover, the protein expressions of BMP2, Runx2, and OSX were also significantly increased in the RSF/5%LAP hydrogel group (Figure 7f). These results, together with the previous research [31], clearly indicated that the introduction of LAP facilitated the osteogenic differentiation of both osteoblasts and BMSCs in the RSF-based hydrogels, resulting in the upregulation of the different osteogenic properties.

The above results are consistent with the literature reports, that is, the incorporation of LAP into various polymer matrices stimulates osteogenic differentiation even in the absence of any osteoinductive factors [31,38,43,44,45]. For example, Xavier et al. reported that after the addition of LAP, the ALP activity and calcium deposition of preosteoblast cells were significantly increased [44]. Zhang et al. evaluated the osteoinductive efficiency with different LAP contents and found that the ALP activity and gene expressions of ALP, Runx2, and OCN increased with the increase in LAP content [45]. Similarly, Miao et al. also found that the protein expressions of Runx2 and Col1 were significantly higher with the addition of LAP [38]. Recently, a novel RSF/LAP 3D porous scaffold with good biocompatibility and osteogenesis capacity was successfully developed in this laboratory [43]. The ALP activity and osteogenic-related gene and protein expression levels (BMP-2, Runx2, and OPN) also increased gradually with the LAP content. Compared with the RSF/LAP scaffold or other LAP-containing materials with fixed geometry reported in the literature, the injectable nature of the RSF/LAP hybrid hydrogel developed in this study allows it to completely fill and fit in irregular defects, such as alveolar bone dehiscence and fenestration, indicating its good application prospects in bone regeneration.

LAP can be decomposed into nontoxic products, including magnesium ions, lithium ions, and silicic acid. These degradation products have been shown to promote the osteogenic differentiation of BMSCs [38]. Magnesium ions can enhance the adhesion and stimulate the osteogenic differentiation of BMSCs [30,46,47,48]. Silicate ion is considered to significantly enhance the proliferation, mineralization nodule formation, and bone-related gene expression of BMSCs [49], while Si(OH)_4_ stimulates collagen type I synthesis and the osteoblastic differentiation of human osteoblasts [50]. Lithium ions can activate the Wnt signaling pathway that regulates osteogenesis via Runx2 activity [51,52,53]. Therefore, LAP is considered to be bioactive and capable of promoting osteogenic differentiation, so it has been widely used for bone tissue engineering [25]. However, the underlying mechanism of the promotion of osteogenic differentiation by LAP has not been fully clarified.

**Figure 8:**
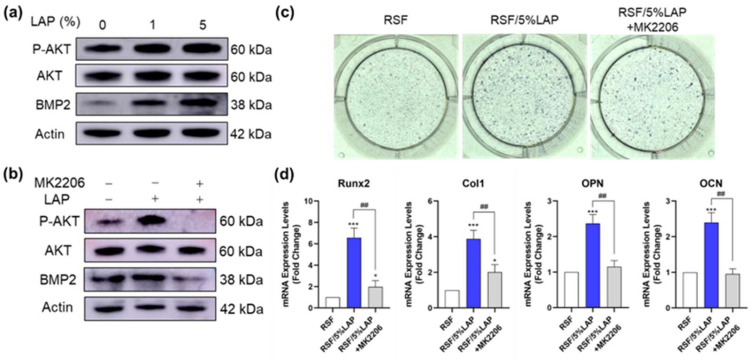
RSF/LAP hydrogels promote osteogenic differentiation of BMSCs by activating the AKT signal pathway: (**a**) expression levels of p-AKT, AKT, BMP2, and β-actin in the hydrogels with different LAP content; (**b**) expression levels of p-AKT, AKT, BMP2, and β-actin in different hydrogel groups. Protein levels of p-AKT and BMP2 were significantly upregulated in the RSF/5%LAP group and suppressed in the RSF/5%LAP + MK2206 group; (**c**) ALP staining on day 7; (**d**) osteogenic-related gene expression on day 7. * *p* < 0.05, *** *p* < 0.001. RSF/LAP hydrogels versus pristine RSF hydrogel: ## *p* < 0.01.

Bone formation is a complex sequence of events, which includes the recruitment, proliferation, and differentiation of BMSCs to an osteoblast lineage [54]. BMSC osteogenesis is the key step in bone regeneration and is affected by several factors, including hormones, growth factors, environmental factors, and microRNAs [33]. AKT-responsive genes are involved in the regulation of several cellular processes, including cell division, autophagy, survival, and differentiation. It is reported that bone formation is promoted by activation of the phosphatidylinositol 3-kinase (PI3K)/AKT signaling pathway [55]. Figure 8a shows that the expression level of p-AKT, as well as the osteogenic marker (BMP-2), increased gradually with the LAP content. To further verify whether RSF/LAP hydrogels promote the osteogenic differentiation of BMSCs by activating the AKT signaling pathway, an inhibitor of AKT (MK2206) was added to the RSF/5%LAP group to block the activation of AKT pathway. It can be seen that the phosphorylation level increased in the RSF/5%LAP hydrogel group compared with the pristine RSF hydrogel group, whereas the p-AKT/AKT level was significantly downregulated after MK2206 treatment (Figure 8b). ALP staining and osteogenic-related gene expression showed consistent results (Figure 8c,d). These findings indicated that LAP in the RSF/LAP hydrogel promoted the osteogenic differentiation of BMSCs through AKT pathway activation. Miao et al. [38] also reported that a blended hydrogel comprising 10% gelatin, 1% alginate, and 2% LAP promoted bone formation by activating the PI3K/AKT signaling pathway. Moreover, the osteogenic properties of the LAP hydrogel were suppressed when an inhibitor of PI3K (LY294002) was used [38]. The results of the present work further confirmed that after the incorporation of LAP, the hydrogels promoted the osteogenic differentiation of BMSCs through AKT pathway activation.

Overall, it can be concluded that the degradation products of the RSF/LAP hydrogel, mainly magnesium ion, lithium ion, and silicate acid, can mediate the activation of the AKT signaling pathway in BMSCs to some extent, which subsequently upregulates osteogenesis-related gene expression. As the RSF/LAP hydrogel can also promote the osteogenic differentiation of osteoblasts [31], the osteogenic ability continues to increase during the terminal differentiation from BMSCs to osteocytes, resulting in accelerated bone repair in the defect area treated by the RSF/LAP hydrogel.

## 4. Conclusions

Herein, an injectable RSF/LAP hybrid hydrogel was developed to further evaluate its biocompatibility and bone regeneration ability in vivo. The subcutaneous implantation of RSF/LAP hydrogels in SD rats resulted in new angiogenesis as well as a mild and controllable inflammatory response. The RSF/LAP hydrogels exhibited an improved bone repairing ability, especially the RSF/5%LAP hydrogel. In addition, the introduction of LAP was found to slow down the degradation of the RSF hydrogel in vivo, which is beneficial for substantial cellular ingrowth and the replacement of the hydrogel by new bones.

Furthermore, in vitro experiments on BMSCs were performed to understand the biological mechanism of the RSF/LAP hydrogels in promoting bone regeneration. The results showed that the RSF/LAP hydrogels improved the adhesion and proliferation of BMSCs. With the increase in LAP content, the ALP activity, mRNA and protein expression of osteogenic markers, and phosphorylation level of AKT increased significantly. Finally, it was found that the inherent osteogenic properties of the RSF/LAP hybrid hydrogel were due to the activation of AKT and suppressed by an AKT inhibitor.

These findings strongly support the feasibility of the injectable RSF/LAP hybrid hydrogel for bone tissue engineering, indicating its promising potential for the treatment of irregular defects, such as alveolar bone dehiscence and fenestration, in future clinical applications.

## Data Availability

The data presented in this study are available in the manuscript itself.

## Supplementary Materials

The following supporting information can be downloaded at: https://www.mdpi.com/article/10.3390/jfb14020086/s1, Figure S1: SEM image combined with EDS elemental mapping of RSF/5% LAP hydrogel; Figure S2: Identification of primary BMSCs; Table S1: Sequences of primers used for PCR amplification.
