# Supplementary material for: Silk–Inorganic Nanoparticle Hybrid Hydrogel as an Injectable Bone Repairing Biomaterial
Source: J Funct Biomater. 2023 Feb 2;14(2):86. doi: 10.3390/jfb14020086 (PMC9966230; doi:10.3390/jfb14020086)
Supplement: Supplementary file 1 [file jfb-14-00086-s001.zip › jfb-2145655-supplementary.pdf]

Supporting Information

Silk–Inorganic Nanoparticle Hybrid Hydrogel as an Injectable Bone Repairing Biomaterial

Liangyan Sun [1,2,†], Minqi Lu [1,3,†], Ling Chen [1,3], Bingjiao Zhao [1,2], Jinrong Yao [1,3], Zhengzhong Shao [1,3], Xin Chen [1,3,*] and Yuehua Liu [1,2,*]

[1]Department of Orthodontics, Shanghai Stomatological Hospital & School of Stomatology, Department of Macromolecular Science, Fudan University, Shanghai, 200433, China

[2]Shanghai Key Laboratory of Craniomaxillofacial Development and Diseases, Fudan University, Shanghai, 200001, China

[3]State Key Laboratory of Molecular Engineering of Polymers, Laboratory of Advanced Materials, Fudan University, Shanghai, 200433, China

* Correspondence: chenx@fudan.edu.cn (X.C.); liuyuehua@fudan.edu.cn (Y.L.)

† These authors contributed equally to this work.

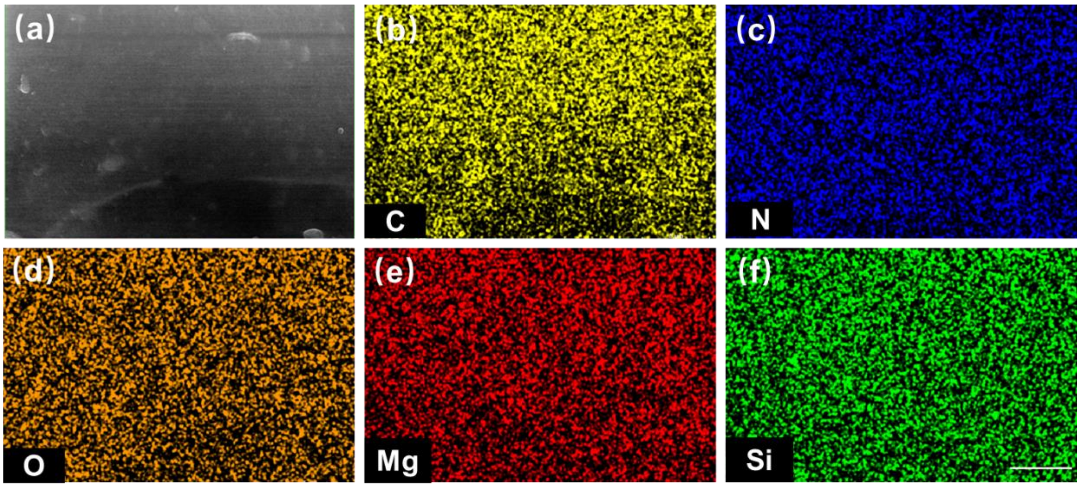

Figure S1. SEM image (a) combined with EDS elemental mapping (b–f) of RSF/5% LAP hydrogel. Scale bar: 5 μm.

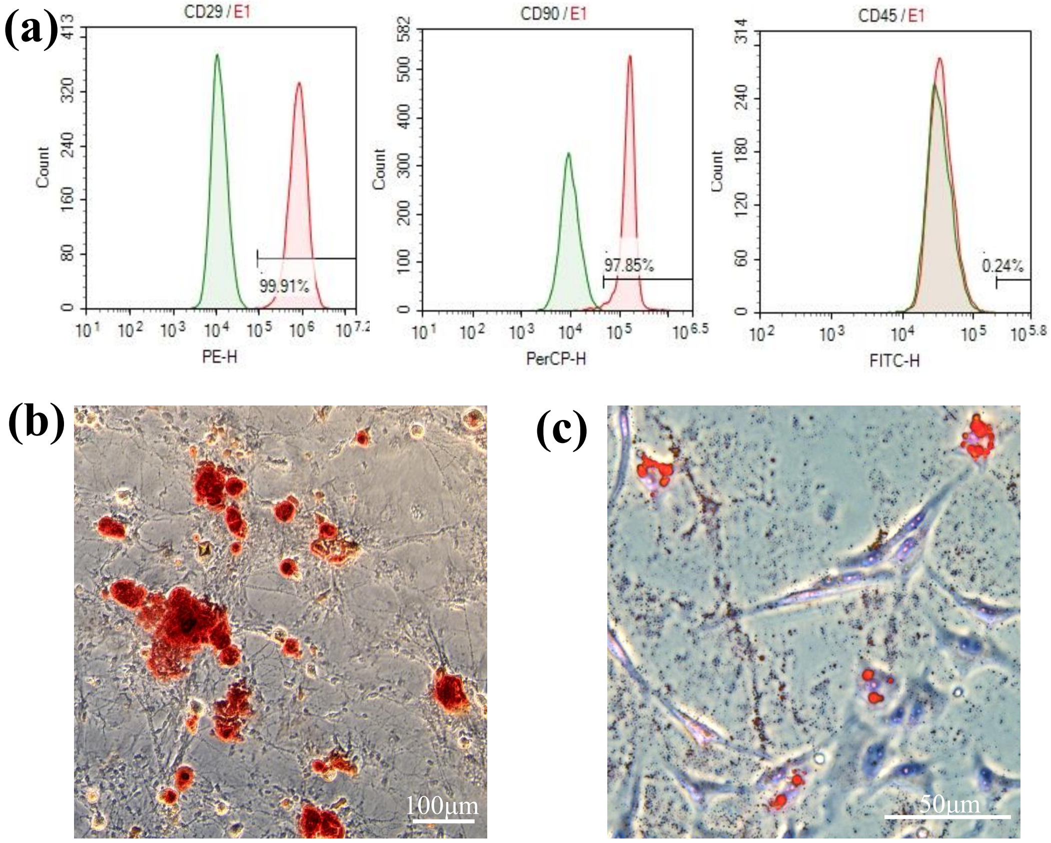

Figure S2. Identification of primary BMSCs. (a) Flow cytometric analysis results of BMSCs showing expression of CD29 (99.91%), CD90 (97.85%), and CD45 (0.24%). (b) Representative images of Alizarin Red staining and (c) Oil Red O staining of BMSCs after osteogenic and adipogenic induction.

Table S1. Sequences of primers used for PCR amplification

Gene name	Forward primer (5′→ 3′)	Reverse primer(5′→ 3′)
Runx2	GCGGTGCAAACTTTCTCCAG	TGCAGCCTTAAATGACTCGG
Col1	GAGGGCCAAGACGAAGACATC	CAGATCACGTCATCGCACAAC
OPN	CTCCATTGACTCGAACGACTC	CAGGTCTGCGAAACTTCTTAGAT
OCN	GGATGACCCCCAAATAGCCC	GCTTGGACACAAAGGCTGC
β-actin	GGCCGGGACCTGACAGACTACCTC	GTCACGCACGATTTCCCTCTCAGC
